# The human papillomavirus E6 protein targets apoptosis-inducing factor (AIF) for degradation

**DOI:** 10.1038/s41598-020-71134-3

**Published:** 2020-08-26

**Authors:** Masaru Shimada, Akio Yamashita, Manami Saito, Motohide Ichino, Takao Kinjo, Nobuhisa Mizuki, Dennis M. Klinman, Kenji Okuda

**Affiliations:** 1 Department of Molecular Biodefense Research, Yokohama City University, Yokohama, 236-0004 Japan; 2 Department of Molecular Biology, Yokohama City University, Yokohama, 236-0004 Japan; 3 Department of Immunology, Yokohama City University, Yokohama, 236-0004 Japan; 4 Division of Morphological Pathology, Department of Basic Laboratory Sciences, School of Health Sciences, University of the Ryukyus, Okinawa, 903-0215 Japan; 5 Department of Ophthalmology and Visual Science, Yokohama City University, Yokohama, 236-0004 Japan; 6 Frederick National Laboratory for Cancer Research, National Cancer Institute, NIH, Frederick, MD 21702 USA

**Keywords:** Cancer, Molecular biology, Molecular medicine

## Abstract

Oncoprotein E6 of high-risk human papillomavirus (HPV) plays a critical role in inducing cell immortalization and malignancy. E6 downregulates caspase-dependent pathway through the degradation of p53. However, the effect of HPV E6 on other pathways is still under investigation. In the present study, we found that HPV E6 directly binds to all three forms (precursor, mature, and apoptotic) of apoptosis-inducing factor (AIF) and co-localizes with apoptotic AIF. This binding induced MG132-sensitive reduction of AIF expression in the presence of E6 derived from HPV16 (16E6), a cancer-causing type of HPV. Conversely, E6 derived from a non-cancer-causing type of HPV, HPV6 (6E6), did not reduce the levels of AIF despite its interaction with AIF. Flow cytometric analysis revealed that 16E6, but not 6E6, suppressed apoptotic AIF-induced chromatin degradation (an indicator of caspase-independent apoptosis) and staurosporine (STS, a protein kinase inhibitor)-induced apoptosis. AIF knockdown reduced STS-induced apoptosis in both of 16E6-expressing and 6E6-expressing cells; however, the reduction in 16E6-expressing cells was lower than that in 6E6-expressing cells. These findings indicate that 16E6, but not 6E6, blocks AIF-mediated apoptosis, and that AIF may represent a novel therapeutic target for HPV-induced cervical cancer.

## Introduction

Certain types of human papillomavirus (HPV) can cause cervical cancer. More than 170 genotypes of HPV (based on HPV L1 gene sequencing) have been identified in proliferative lesions of the skin or mucosa; of these, more than 40 are sexually transmitted^1^. HPVs that infect the genital mucosal epithelium are classified as high- or low-risk types. Infection with high-risk HPV can cause precancerous lesions that may progress into invasive tumors, while infection with low-risk types can cause skin warts but not cancer^2^. At least 12 genotypes are defined as high-risk types, in which HPV16 and HPV18 have the highest cancer risk, accounting for approximately 70% of cervical cancers^3^. By modulating a range of cellular pathways, including cell cycle regulation and apoptosis, the combined activity of the two major viral oncoproteins, E6 and E7, result in cell immortality and malignant tumors^4, 5^.

E6 in high-risk HPV types is a key oncoprotein that induces cell immortalization and malignancy. The E6 oncoprotein binds to the E6-associated protein (E6AP, a cellular E3 ubiquitin ligase) to form an E6/E6AP complex^6, 8^. P53 is one of the major targets of this complex. By targeting the p53 tumor suppressor for ubiquitination and proteasomal degradation, this complex interferes with p53-mediated cell cycle arrest, thus enabling tumors to survive and proliferate^6^. E6 from high-risk HPV types, such as HPV16, can also support human carcinogenesis via a p53-independent pathway^7^. For example, the E6/E6AP complex can induce the degradation of the transcriptional repressor NFX1-91, leading to increased transcription of human telomerase reverse transcriptase (hTERT), telomere shortening, and cell proliferation^8, 9^.

E6 has been identified to interact with many cellular proteins which may support its oncogenic activity. Some of these proteins also bind to the E6 protein of low-risk HPV types. For example, E6 protein from both high- and low-risk HPVs can bind to Bcl-2 homologous antagonist killer (Bak) and increase its rate of proteolytic turnover through the E6AP-proteasome pathway^10^. However, high-risk E6 binds more strongly to p300/CBP^11^, and only high-risk E6 protein binds to E6BP/ERC-55^12^, MCM7^13^, c-Myc^14^, and paxillin^15^, which are strongly associated with cell transformation and apoptosis.

Two major apoptotic pathways exist: the caspase-dependent and caspase-independent pathways. In the caspase-dependent pathway, an extrinsic death program activated by tumor necrosis factor (TNF) receptors forms an intracellular death-inducing signaling complex. In contrast, the intrinsic apoptotic program, which is activated by death signals such as cellular stress, is regulated predominantly via mitochondria in a caspase-independent manner. These stimuli induce mitochondria to release apoptosis-related factors, such as AIF (apoptosis-inducing factor), endonuclease G, Omi/HtrA2, Smac/DIABLO and cytochrome c^16–18^. It has been shown that Omi/HtrA2, Smac/DIABLO and cytochrome c induce apoptosis predominantly via the caspase-dependent pathway, while AIF and endonuclease G induce apoptosis by the caspase-independent pathway^19^.

AIF is a flavoprotein that supports cell viability as a mitochondrial oxidoreductase, but can also mediate cell death through its pro-apoptotic nuclear activity^20, 21^. Human AIF has three forms: precursor, mature and apoptotic forms. The precursor form of AIF is expressed as a 613 amino acid and contains two nuclear leading sequences (NLS) in each FAD domain (Flavin adenine dinucleotide) and an N-terminal mitochondrial localization sequence (MLS)^22^ (Fig. 3a). After import into the mitochondria, the precursor form of AIF is cleaved at the N-terminal 54 residues to generate the mature form (Δ54AIF). The mature form is inserted into the inner mitochondrial membrane, where it incorporates a FAD cofactor and folds into three structural domains^23^. After exposure to an apoptotic insult, the mature form of AIF is cleaved at residue 102 to yield a soluble, apoptotic form (Δ102AIF). The apoptotic form of AIF is translocated first to the cytoplasm and then to the nucleus, inducing apoptosis via chromatin condensation and large-scale DNA fragmentation^21, 24^. The mature form of AIF is composed of three structural domains: the FAD-binding, nicotinamide adenine dinucleotide (NADH)-binding, and C-terminal domains^23^. Interestingly, AIF has NADH oxidase activity *in vitro*^25^ and is able to protect against certain forms of oxidative stress *in vivo*^26^. The protection afforded by AIF relies in part on maintaining the expression and activity of complex I of the electron transport chain^20^. Loss of AIF in heart or brain tissue can lead to a life-threatening loss of mitochondrial integrity^27, 28^. More recently, mutations in human AIF have been implicated in several diseases, such as mitochondrial human disorders, early onset severe neuromuscular disorders, deafness, and cognitive impairment^29–31^.

This study demonstrates that E6 (Supplementary Fig. S4 online) from both high- and low-risk HPV types bound to AIF, while only high-risk E6 was able to induce the degradation of AIF, resulting in the inhibition of AIF-mediated apoptosis.

## Results

### HPV16 E6 protein binds to AIF

E6 from high-risk HPV plays an important role in HPV-induced cancer; therefore, we detected cellular proteins that interact with HPV16 E6 (16E6) using affinity purification mass spectrometry. 293TT cells were transfected with p16E6-SBP and HPV16 E6-binding cellular proteins were pulled down, purified, and analyzed by mass spectrometry. pGFP-SBP was used as a control. Most of the proteins pull-downed by 16E6-SBP were also found in that by GFP-SBP. Some well-known 16E6-interating proteins, such as E6AP and P53, were only defined in the proteins pull-downed by 16E6-SBP, but not by GFP-SBP, indicating the specificity of pull-down experiments, and SBP peptide can be used as a protein tag for the experiments (data not shown). AIF was one of newly defined 16E6-interacting protein, which were found neither in the proteins pull-downed by GFP-SBP, nor in a published database (Virus-Mint, https://amp.pharm.mssm.edu/Harmonizome/resource/Virus+MINT)^32^. Given the importance of E6 in inducing cervical cancer and the importance of AIF in mediating caspase-independent apoptosis^19^, we hypothesize that E6 may affect AIF. Therefore, the relationship between E6 and AIF was analyzed in greater detail.

**Figure 1:**
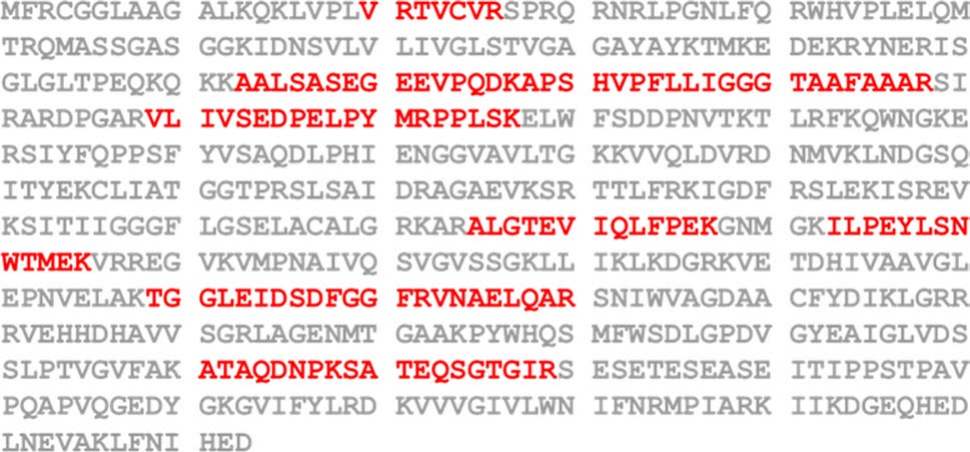
AIF peptide sequences identified by LC/MS–MS analysis. The figure shows the amino acid sequence of AIF with the overlapping peptide sequences captured by HPV16 E6 highlighted in red.

**Figure 2:**
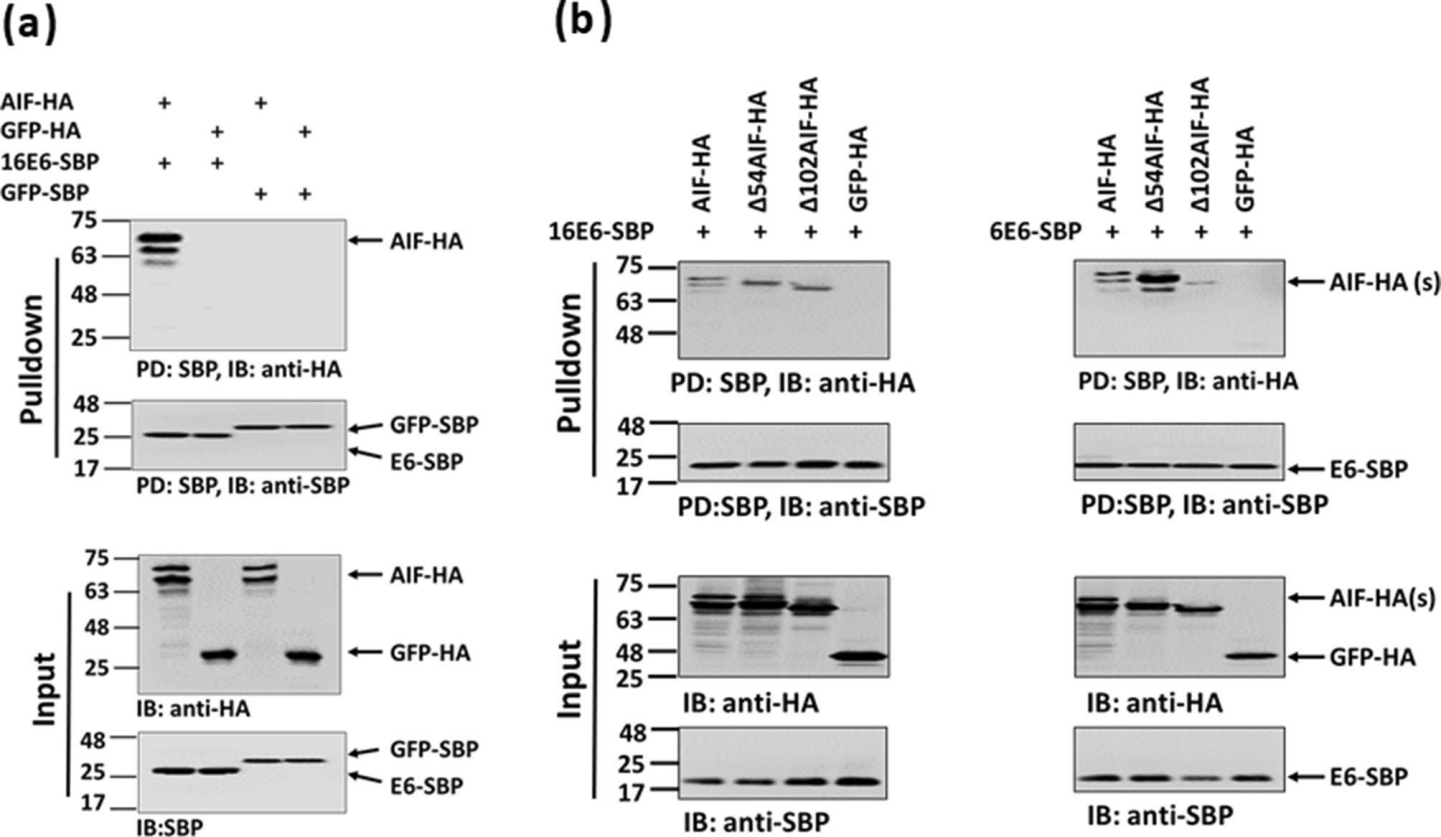
Binding of E6 to AIF. Expression plasmids (shown at the top) were co-transfected into 293TT cells by using PEI. Then, proteins in the cell lysates were pulled down using Streptavidin Mag Sepharose and immunoblotted with anti-HA-tag and anti-SBP antibodies. (**a**) Confirmation of HPV16 E6 binding to AIF. (**b**) Confirmation of the binding of E6 from HPV16 and HPV6 to three AIF forms. Numbers on the left are the molecular masses (in kDa). Five independent experiments were performed and one of them is shown. PD: pulldown; IB: immunoblot.

The results showed that seven of the peptides isolated from p16E6-SBP-transfected cells had amino acid sequences that overlapped with that of AIF (isoform 1 and 3), covering 21% of the polypeptide sequence for both isoform 1 and 3 (Fig. 1). AIF has 6 isoforms classified as isoform 1 ~ 6 (UniProt, https://www.uniprot.org/). Compared to isoform 1 and 3, isoform (2, 4 ~ 6) have proximately 50% deletion in length, which may effect on the protein-binding capacity of the AIF. To confirm the hypothesis that E6 interacts with AIF, combinations of the p16E6-SBP, pAIF-HA, pGFP-HA, and pGFP-SBP plasmids were co-transfected into 293TT cells, and the interacting proteins were pulled down using Streptavidin Mag Sepharose and detected with anti-HA-tag and anti-SBP-tag antibodies. As shown in Fig. 2a and Supplementary Fig. S1a online, AIF-HA was pulled down from cells co-transfected with pAIF-HA and p16E6-SBP, but not any other combination.

We then compared the effect of transfecting 293TT cells with plasmids expressing E6 from a high- and low-risk HPV type (p16E6-SBP and p6E6-SBP, respectively). Cells were co-transfected with E6-expressing plasmid and AIF (pAIF-HA) or one of its variants, pΔ54AIF-HA, or pΔ102AIF-HA. Plasmid pAIF-HA expresses precursor AIF, pΔ54AIF-HA expresses mature AIF, and pΔ102AIF-HA expresses apoptotic AIF. As shown in Fig. 2b and Supplementary Fig. S1b online, both 16E6 and 6E6 bound to AIF and its variants. These findings establish that E6s from both high- and low-risk HPV bind to AIF.

### Identification of the AIF-binding domain

**Figure 3:**
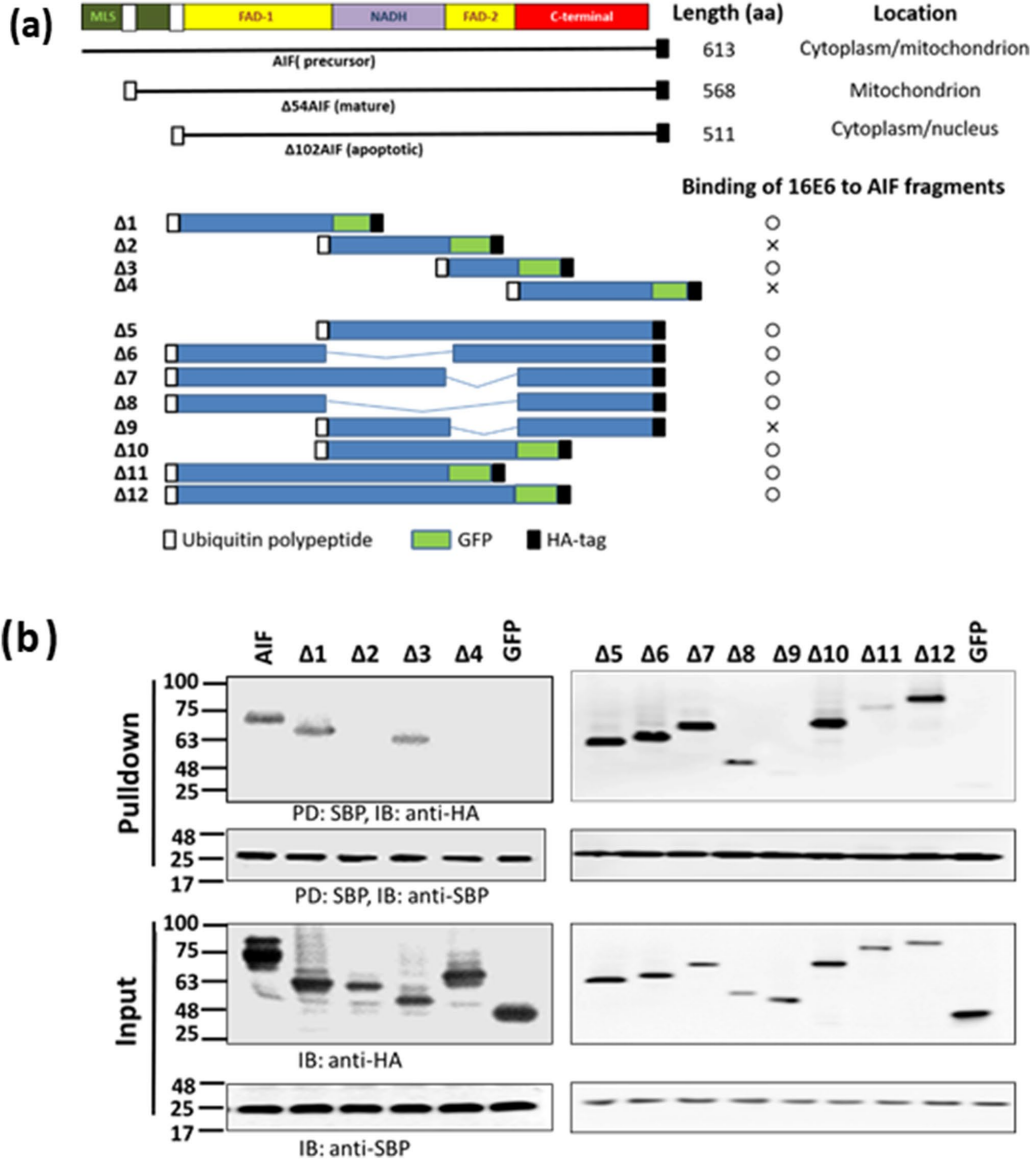
E6 binds to the FAD domain of AIF. (**a**) List of schematics of AIF and the various constructs used in the study. In all constructs, except full length AIF, AIF was fused with an ubiquitin polypeptide at the N-terminus, and all AIF proteins had an HA-tag at the C-terminus. In some constructs (Δ1, Δ2, Δ3, Δ4, Δ10, Δ11, and Δ12), AIF was fused to GFP at its C-terminus. The results of 16E6 binding to AIF fragments were transferred from (**b**). A circle (○) indicates binding, and × indicates no binding. MLS: mitochondrial localization sequence; FAD: flavin adenine dinucleotide-binding domain; NADH: nicotinamide adenine dinucleotide-binding domain; C-terminal: C-terminal domain. (**b**) 293TT cells were co-transfected with p16E6-SBP and AIF or variant expression plasmids by using PEI, and then the proteins in the cell lysates were pulled down with Streptavidin Mag Sepharose and immunoblotted with anti-HA-tag and anti-SBP antibodies. Three independent experiments were performed and one of them is shown. Numbers on the left are molecular masses (in kDa). PD: pulldown; IB: immunoblot.

AIF contains a FAD-binding, NADH-binding domain, and C-terminal domain (Fig. 3a). Each of these domains was subcloned and fused to a GFP protein with an HA-tag at the C-terminus. Cells were co-transfected with p16E6-SBP plus one of these domain-restricted plasmids. The SBP pulldown studies showed that the E6 protein bound to the FAD domain, but not the NADH or C-terminal domains (Fig. 3b, left panel and Supplementary Fig. S2 online). To confirm this finding, additional constructs were prepared in which one or two domains of AIF were deleted (Fig. 3a). In some constructs (Δ10, Δ11, and Δ12), these domain(s) were fused to GFP to improve protein expression and detection, since expression of the protein fragment alone was too low to be detected by Western blotting when the C-terminal domain was deleted (data not shown). The results confirmed that the E6 protein selectively bound to constructs containing the either of N- or C-terminal parts of AIF FAD domain (Fig. 3b and Supplementary Fig. S2 online, right panel). Of note, protein expression levels were remarkably lower when the C-terminal domain was deleted, indicating that the C-terminal domain may be important for stability (Fig. 3a).

### E6 co-localizes with the apoptotic form of AIF

**Figure 4:**
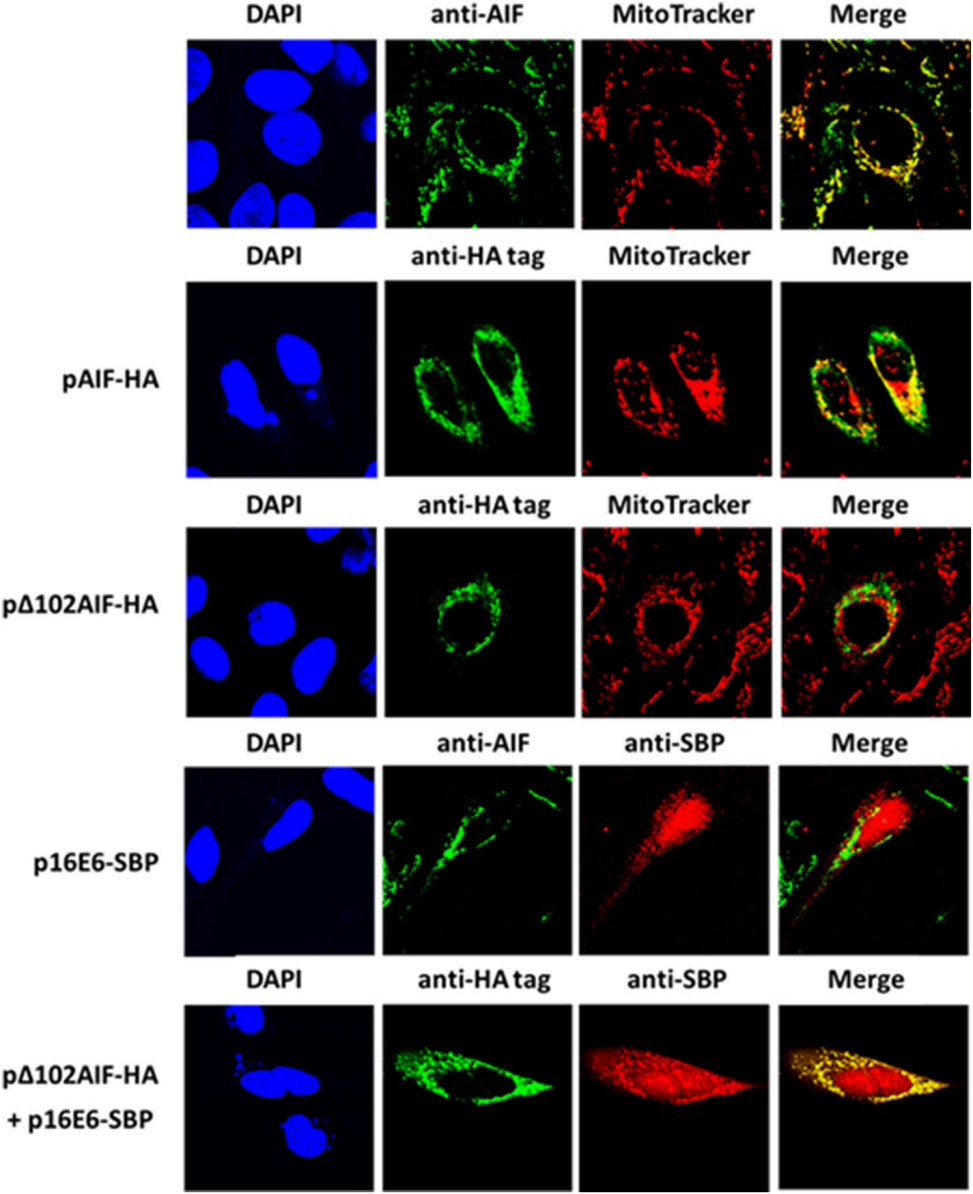
Co-localization of E6 with Δ102AIF. U2OS cells were transfected with p16E6-SBP, pAIF-HA, or pΔ102AIF-HA alone or in combination by using PEI for 20 h. Then, the cells were stained with MitoTracker Red (red), DAPI (blue), anti-AIF antibody (green), anti-HA-tag antibody (green) and/or anti-SBP-tag antibody (red). Yellow indicates co-localization of proteins labeled with green and red. Three independent experiments were performed and one of them is shown. Fifty of positive cells were counted for each staining.

There are three forms of AIF, precursor, mature, and apoptotic (Fig. 3a). While, when expression plasmids encoding one of the three form AIF gene transfected to cells, the protein of the latter two forms cannot translocate to the mitochondria because they lack a mitochondrial localization sequence (MLS; Fig. 3a). In healthy cells, most AIF exists as the mature form. We detected endogenic AIF in healthy cells using an anti-AIF antibody and found that almost all of the endogenic AIF was localized to the mitochondria (stained with MitoTracker), and not in other parts of the cell (Fig. 4, top panel). Localization of the precursor and apoptotic forms of AIF was monitored by transfecting U2OS cells with the pAIF-HA and pΔ102AIF-HA plasmids. AIF protein was detected by staining with an anti-HA-tag Ab. Most of the transfected AIF was detected in the mitochondria, a small amount was detected in the cytosol, and none was detected in the nucleus in pAIF-HA-transfected cells (Fig. 4, second panel). In contrast, in pΔ102AIF-HA-transfected cells, most AIF was detected in the cytosol (Fig. 4, third panel).

To explore whether E6 co-localized with AIF, U2OS cells were transfected with p16E6-SBP. 16E6 was present in both the nucleus and cytosol, and E6 did not co-localize with endogenic AIF (most of which was the mature form) in the cytosol (Fig. 4, fourth panel). When cells were co-transfected with p16E6-SBP and pΔ102AIF-HA, co-localization of 16E6 and Δ102AIF was observed in the cytosol, demonstrating that 16E6 co-localized with the apoptotic form of AIF in the cytosol.

### E6 from HPV16 causes AIF to be degraded by the proteasome

**Figure 5:**
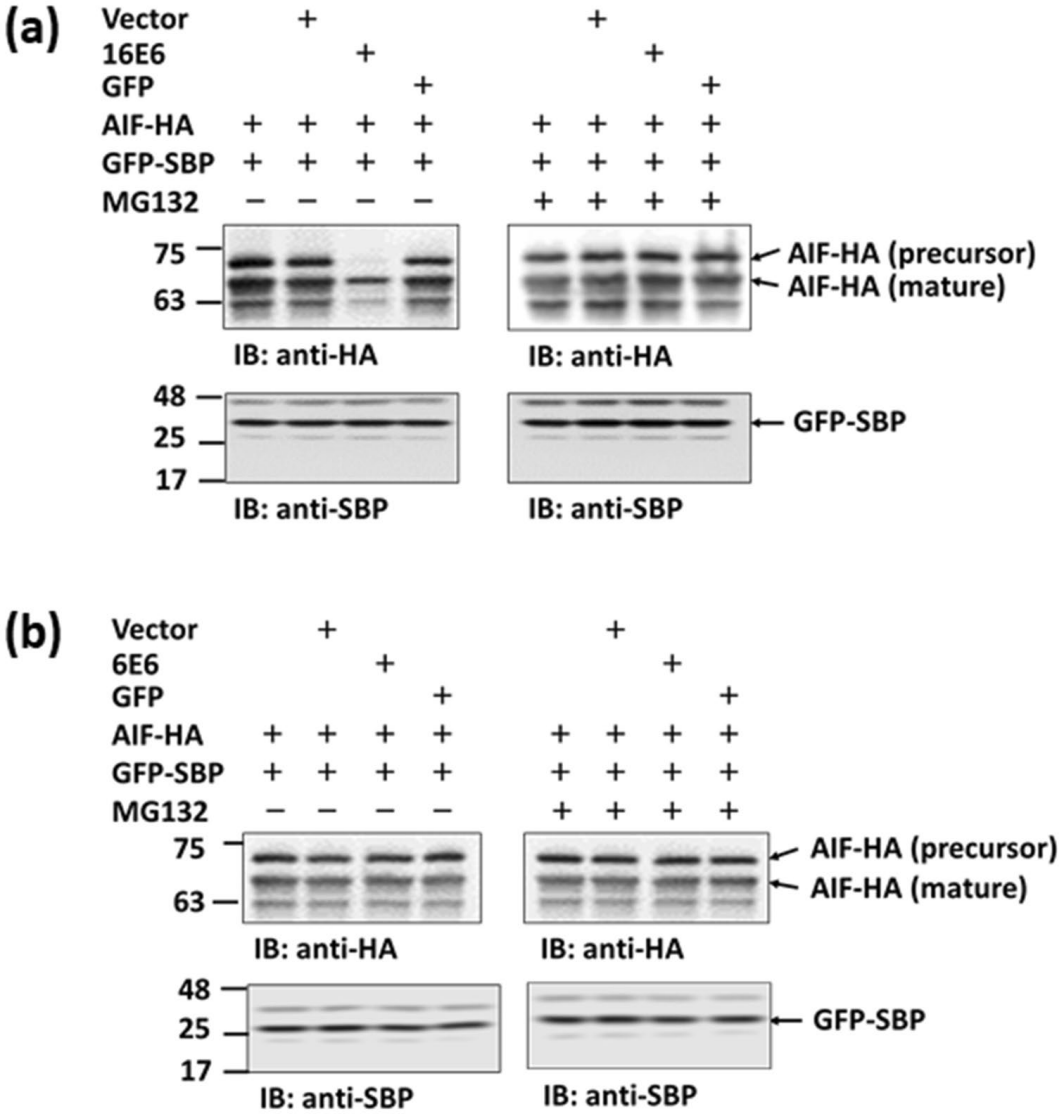
Proteasomal degradation of AIF induced by E6. 293TT cells co-transfected with pAIF-HA and p16E6 (**a**) or p6E6 (**b**) by using PEI. After 24 h, the cell lysate was immunoblotted with anti-HA-tag and anti-SBP antibodies. pGFP-SBP was used as a transfection internal control. In some samples, cells were treated with MG132 12 h prior to cell harvest. Five independent experiments were performed and one of them is shown. Numbers on the left are molecular masses (in kDa). IB: immunoblot.

To study the effect of E6 on AIF, 293TT cells were co-transfected with p16E6 or p6E6 and pAIF-HA, and the expression of each form of AIF was monitored. The results showed that the expression of AIF was strongly reduced by 16E6 but not 6E6 (Fig. 5a,b and Supplementary Fig. S3 online). 16E6-induced reduction was inhibited by the addition of the proteasome inhibitor MG132.

### E6 inhibits AIF-dependent chromatin degradation

**Figure 6:**
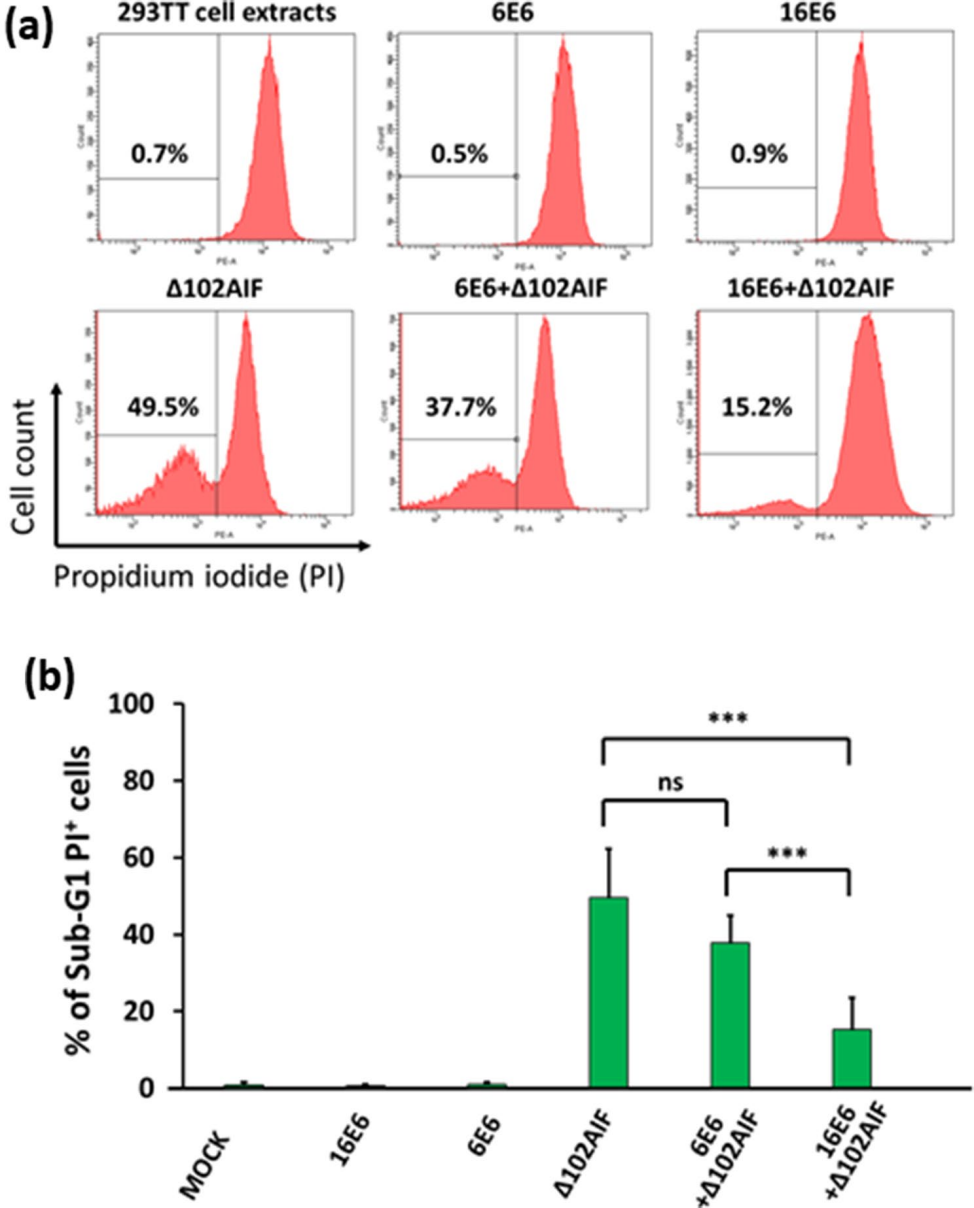
Inhibition of AIF-induced chromatin degradation by HPV16 E6. 293TT cells were transfected with p16E6, p6E6, and/or pΔ102AIF expression plasmids. Then, cell lysates from the transfected cells were incubated with nuclei isolated from HeLa cells and stained with propidium iodide (PI). Chromatin degradation was assessed by flow cytometry and quantified by sub-G1 population gating. (**a**) Representative flow cytometric panels are shown. (**b**) The percentages of sub-G1 PI-positive cells from three independent experiments performed with five samples were analyzed, *** indicates a significant difference between the two groups. ns, no significant difference. Statistical analyses were performed by Krushal-Wallis test with Steel–Dwass test.

To explore the effect of E6 on AIF-induced chromatin degradation, HeLa nuclei were incubated with cytosolic extracts from 293TT cells transfected with p16E6, p6E6, and pΔ102AIF alone or in combination. Chromatin degradation was assessed by flow cytometric detection of PI-stained cells. Due to self-destruction and fragmentation, chromatin size of the apoptotic cells is smaller than normal G1 cells which has diploid DNA. We defined the high and low PI-density peak as G1 nuclei and apoptotic nuclei, respectively. Total 30,000 nuclei were counted and % of the apoptotic nuclei was calculated from the proportion in total nuclei. Background levels of chromatin degradation were < 1% in nuclei exposed to normal-, 16E6-, or 6E6-transfected extracts (Fig. 6a). When the nuclei were treated with Δ102AIF-extract, chromatin degradation rose to 49.5%. This degradation was significantly inhibited (by > 50%; *p* < 0.05) when 16E6 extract was added with the Δ102AIF-transfected extract, but not when 6E6 extract was added (*p* > 0.05; Fig. 6a,b).

### 16E6 inhibits AIF-inducing apoptosis

**Figure 7:**
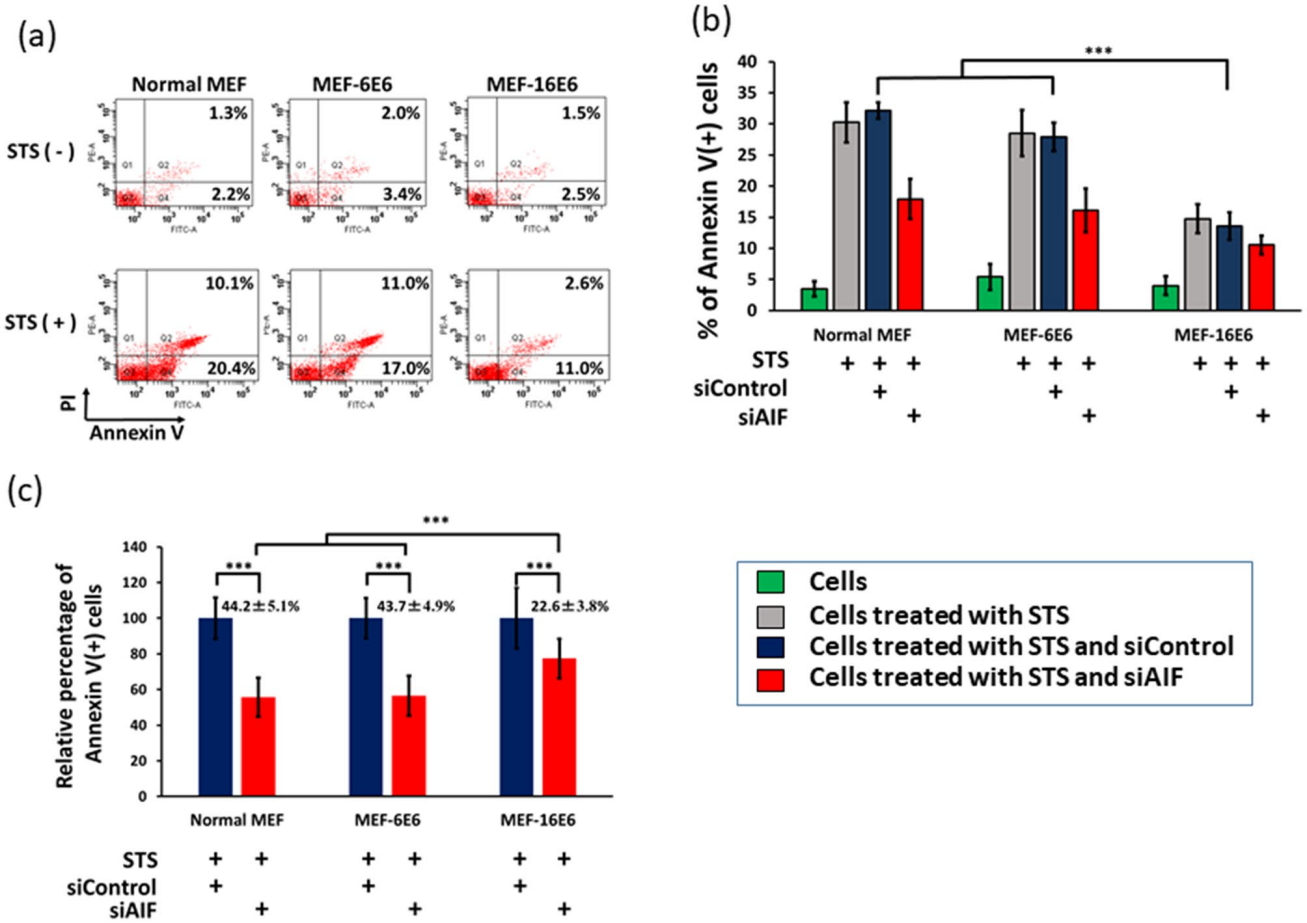
E6 from HPV16 inhibits AIF-mediated apoptosis in MEF cells. (**a**) Normal MEF cells, HPV16 E6-expressing (MEF-16E6) and HPV6 E6-expressing (MEF-6E6) MEF cells were treated with or without STS (a protein kinase inhibitor). Four hours after STS stimulation, cells were stained with PI and FITC-Annexin V and analyzed by flow cytometry. Annexin V + PI- and Annexin V + PI + cells were defined as early and late apoptotic cells, respectively. (**b**) Normal MEF, MEF-16E6, and MEF-6E6 cells were transfected with siControl or siAIF twice with a 6 h interval and then treated with STS for 4 h. Cells were stained with PI and FITC-Annexin V and analyzed by flow cytometry. Normal MEF cells were included as a negative control. Raw data were shown in (**b**). The relative effect of  siAIF compared with siControl was shown in (**c**). Each sample was analyzed in quadruplicate. Three independent experiments were performed and one of them is shown. *** indicates a significant difference between the groups (*p* < 0.05). Statistical analyses were performed by the Mann–Whitney U test for two group comparison and by Krushal-Wallis test with Steel–Dwass test for multi-group comparison.

Since staurosporine (STS, a protein kinase inhibitor) is able to induce both of caspase-dependent and caspase-independent apoptosis^33^, we assessed the effect of E6 on STS-induced apoptosis. For this we used MEF cell lines stably expressing HPV16 E6 (MEF-16E6) and HPV6 E6 (MEF-6E6). Annexin V-FITC, which captured apoptosis inducing phosphatidylserine translocation^34^, positive cell defined as apoptotic cells^35^. Treatment with STS significantly increased the apoptotic cell number in normal MEF cells (Annexin V positive cells, 3.5% vs. 30.5%, *p* < 0.05) (Fig. 7a). The apoptotic fraction of MEF-16E6 cells (Annexin V^+^ cells) was significantly suppressed compared to that of the normal MEF cells (13.6% vs. 30.5%, *p* < 0.05) and MEF-6E6 cells (13.6% vs. 28.0%, *p* < 0.05).

To explore the role of AIF in 16E6-mediated inhibition of apoptosis, normal MEF, MEF-16E6, and MEF-6E6 cells were transfected with siRNA targeted with AIF (siAIF) or control siRNA (siControl) and treated with STS. Note that siAIF inhibited AIF expression by more than 90% (data not shown). Transfection of siAIF significantly reduced the number of apoptotic cells in normal MEF and MEF-6E6 cells (44.2% and 43.7% reduction, respectively). On the other hand, MEF-16E6 cells showed lesser siAIF effect (22.6% reduction.) (Fig. 7b,c). This demonstrates that AIF plays an important role in 16E6-mediated inhibition of apoptosis.

## Discussion

This study demonstrated that HPV E6 binds to AIF, altering its activity. It also showed that E6 binds to the FAD domain of AIF and that E6 from high-risk, but not low-risk, HPV induces AIF degradation via the proteasome, and high-risk HPV E6 thereby suppresses AIF-mediated apoptosis. These results suggest that AIF-mediated apoptosis plays an important role in high-risk E6-induced cervical cancer.

E6 from high-risk plays a critical role in carcinogenesis by affecting cellular transformation and immune response^36^. However, as E6 lacks enzymatic activity, it exerts its effects via contact with numerous cellular proteins^7, 37, 38^. Among the proteins that interact with E6, p53 is one of the most important^7^. p53 is a transcription factor that regulates the expression of genes involved in the cell cycle, DNA repair, and apoptosis. It has been reported that E6 from high-risk HPV16 binds to the conserved LXXLL motif of E6AP, a cellular protein with E3 ubiquitin protein ligase activity^39, 40^. The E6/E6AP complex binds to p53, resulting in its ubiquitination and proteasomal degradation. We found that E6 from both high- and low-risk HPV types (HPV16 and HPV6, respectively) bound to all of three forms of AIF (Fig. 2 and Supplementary Fig. S1 online). Further analysis showed that the binding site(s) were located in the FAD domain of AIF (Fig. 3). However, no LXXLL motif was identified in this domain (data not shown). We also found that high-risk E6 could induce the degradation of AIF, and that this activity was inhibited by the proteasome inhibitor MG132. Therefore, we hypothesize that E6 may form a complex with some other protein(s), and then bind to AIF.

There are three forms of AIF, precursor, mature, and apoptotic. All three forms contain FAD-binding, NADH-binding, and C-terminal domains. It has been shown that AIF has a calpain- and/or cathepsin-cleavage site, Hsp70-binding site, Cyp A-binding site^23^, and two DNA-binding sites^21^. To identify the domain to which E6 binds, we fused each domain of AIF to an HA-tagged GFP protein. This was necessary because the expression levels of constructs expressing certain domains were too low to conduct a SBP-pulldown assay. The results showed that the C-terminus may play a role in AIF protein stability. To confirm that E6 bound to the AIF FAD domain, we examined E6 binding using constructs in which one or two of the AIF domains were deleted. These studies confirmed that E6 bound to the FAD domain of AIF (Fig. 3, Supplementary S2 online).

The E6 protein is composed of 150 amino acids with two zinc-binding domains, one E6AP-binding domain, and one PDZ protein-binding domain^41^. Although E6 plays an important role in HPV-induced cervical cancer, the E6 protein itself has no enzymatic activity. E6 can bind to proteins directly via its LXXLL motif and PDZ domain and indirectly by forming a complex with the ubiquitin ligase E6AP^42, 43^. The E6/E6AP complex binds to numerous other proteins, inducing their ubiquitination and subsequent proteasome-mediated degradation^44^. White et al. analyzed the host proteins that interact with E6 from alpha and beta genera HPV^38^. They reported that AIF was bound by the E6 protein from HPV8 and HPV17 but not the E6 protein from other HPV types. In this study, AIF bound to E6 from both high- (type 16) and low-risk (type 6) HPV. This difference may reflect the greater sensitivity of the transient expression system used in the current study. Indeed, many more E6-binding proteins were detected using transiently transfected 293TT cells than using White’s stably infected cells (data not shown)^45^. Over-expression system is able to increase the sensitivity for E6 binding protein screen, on the other hand, it also can increase the possibility of non-specific interactions. It should very carefully interpret the data when over-expression system was used. In this study, we used GFP-SBP as a negative control, since the expression of GFP-SBP was more than fivefold higher than that of 16E6-SBP. Actually most of proteins pull-downed by 16E6-SBP was also observed in the proteins pull-downed by GFP-SBP. However, some well-known E6-binding proteins, such as E6AP and P53, were only observed in the proteins pull-downed by 16E6-SBP, but not by GFP-SBP (data not shown). For example, all isoforms (I ~ III) of E6AP and all isoforms (1–9) of p53 (UniProt, https://www.uniprot.org/) were defined in the proteins pull-downed by 16E6-SBP (21% of amino acid coverage for E6AP and 3% for p53), indicating that SBP peptide-fusion protein can be used for pull-down experiments. Furthermore, SBP peptide-fusion protein also has been used in our previous studies^46–48^.

E6 can alter cancer susceptibility through its interactions with p53 and proteins in other pathways^49^. For example, E6 can bind to and induce the degradation of various apoptosis-related proteins, including C-myc^50^, procaspase 8^51^, Fas-associated death domain (FADD)^52^, and tumor necrosis receptor 1 (TNF R1)^53^, and alter the transcription of survivin^54^. Leverrier et al. reported that E6 can inhibit AIF migration from the mitochondria to the cytosol by binding to and inducing the degradation of Bak^55^. However, our data demonstrate that E6 can bind to AIF and promote its degradation, thereby inhibiting AIF-mediated apoptosis. AIF look like is a network hub protein, at least 119 proteins has been reported to interact with AIF (gene database, https://www.ncbi.nlm.nih.gov/gene/9131), indicating that AIF may be a multi-functional protein. When 293TT cells were co-transfected with p16E6 and pAIF-HA, precursor AIF was more degraded than mature AIF (Fig. 5a and Supplementary Fig. S3a online). This could be explained by the fact that E6 localizes to the cytosol and nucleus, but not to the mitochondria (Fig. 4); thus, E6 effects less on the degradation of the mature form of AIF (located in mitochondria).

To explore the effect E6 on AIF-induced chromatin degradation, HeLa cells were used. We choose HeLa cells in this study since HeLa cells have been used for chromatin assay in many published studies^56^. In the next study, HPV16 harboring cells, such as CaSki or SiHa cells, also should be examined.

In this study, we successfully used a 38 amino acid peptide (SBP) as a tag at the c-terminus of E6. However, degradation of p53 and AIF was not observed when using the 16E6-fusion protein, but observed by native 16E6 (Fig. 5a and Supplementary Fig. S3a online), indicating that E6 with the large peptide may loss some bioactivity. On the other hand, degradation of AIF was also not observed when using 16E6 fused with short peptide such as HA tag and Flag tag (with 9 and 8 amino acids, respectively), indicating E6 protein itself is very sensitive and complex. Based on our immunostaining experiments (Fig. 4), we found most of endogenous AIF existing as mature form in cells, and remarkable degradation of endogenous AIF was not observed when the 293TT cells were transfected with p16E6 (data not shown).

In summary, this work demonstrates that HPV E6 can bind to AIF, and high-risk HPV E6 can induce the degradation of AIF and inhibit AIF-mediated apoptosis. These findings suggest that AIF may represent a novel target for inhibiting the development of HPV-induced cancer.

## Materials and methods

### Cell lines

293TT is a derivative of the 293 T cell line (a human kidney epithelia cell line) containing multiple copies of the SV40 large T gene, and an SV40 origin-containing plasmid can replicate in these cells. U2OS is a human epithelial cell line from osteosarcoma with wild type p53. Cells were cultured in Dulbecco’s modified Eagle’s medium (DMEM; Wako Corp, Tokyo, Japan) containing 10% fetal bovine serum (FBS) at 37 °C in 5% CO_2_. Mouse embryonic fibroblasts (MEFs, CF-1 strain) were purchased from ATCC (Manassas, VA, USA) and MEF cells stably expressing E6 from HPV16 (MEF-16E6) and HPV6 (MEF-6E6) were constructed as previously described^57^. The cells were cultured in DMEM medium containing 15% FBS at 37 °C in 5% CO_2_.

### Plasmids

The mammalian expression plasmid pCAGGS (GenBank Access No. LT727518) was used in this study. All transgene fragment was inserted into *Eco*R I site of pCASSG vector using homologous recombination cloning method (Gibson assembly system, New England Biolab. Tokyo, Japan). The E6 genes of HPV16 and HPV6 were synthesized as a human codon optimized form (Supplementary Table S1 online) and amino acid sequences of high risk and low risk HPV E6 were aligned in Supplementary Figure S4 using ClustalW software (Version 2.1, https://clustalw.ddbj.nig.ac.jp/). The cDNAs for full length AIF, the mature form (Δ54AIF), and apoptotic form (Δ102AIF) were amplified from a HeLa S3 cDNA library (Agilent Technologies, Inc., Santa Clara, CA, USA) by PCR. Since a methionine residue at the amino terminus of AIF could potentially interfere with the binding of AIF to other proteins^58^, Δ54AIF and Δ102AIF were expressed as fusions with an ubiquitin polypeptide at the N-terminus^59^. E6 from HPV16 and HPV6, full-length AIF, ubiquitin polypeptide-fused Δ54AIF, ubiquitin polypeptide-fused Δ102AIF, and green fluorescent protein (GFP) were fused with a streptavidin-binding protein (SBP)-tag at the C-terminus, and subcloned into the pCAGGS plasmid to produce p16E6-SBP, p6E6-SBP, pAIF-SBP, pΔ54AIF, and pΔ102AIF, and pGFP-SBP, respectively. Full-length AIF, ubiquitin polypeptide-fused Δ54AIF, and ubiquitin polypeptide-fused Δ102AIF were amplified with primers containing an HA-tag at the C-terminus and then sub-cloned into pCAGGS to construct pAIF-HA, pΔ54AIF-HA, and pΔ102AIF-HA, respectively. SBP DNA was synthesized by GenScript Corp. (Tokyo, Japan). The DNA sequences of SBP and HA were 5ʹ-GACGAGAAAACCACCGGCTGGCGGGGAGGCCACGTGGTGGAAGGGCTGGCAGGCGAGCTGGAACAGCTGCGGGCCAGACTGGAACACCACCCCCAGGGCCAGAGAGAGCCT-3ʹ, and 5ʹ-TACCCATACGATGTTCCAGATTACGCT-3ʹ, respectively.

To identify the binding domain(s) of AIF, AIF fractions were fused to an ubiquitin polypeptide at the N-terminus and an HA-tag at the C-terminus, and then subcloned into pCAGGS (Fig. 3a). In some constructs, AIF was fused to an ubiquitin polypeptide at the N-terminus and to GFP-HA at C-terminus after subcloning into the pCAGGS vector. More detail information is available from Supplementary Table S1 online and corresponding author.

### Small interfering RNA (siRNA)

siControl and siAIF were synthesized by Japan Qiagen, Inc. (Tokyo, Japan). The siRNA sequences were as follows: siControl, 5ʹ-UUCUCCGAACGUGUCACGU-3ʹ; and siAIF, 5ʹ-GCGAUUCAAACAGUGGAAU-3ʹ, 5ʹ-CACAGUGGAAUUGGCAAAC-3ʹ, and 5ʹ-UGGUGGCUUCCGGGUAAAU-3ʹ^60^. Normal MEF, MEF-16E6, and MEF-6E6 cells were cultured on a plate one day before transfection with siControl or siAIF using RNAiMAX (ThermoFisher Scientific, Yokohama, Japan) at a final concentration of 50 nM twice at 6 h intervals, according to the manufacturer’s instructions. Forty-eight hours post-transfection, the cells were treated with STS for 4 h and then analyzed by flow cytometry.

### Antibodies and sepharose beads

The following antibodies were used as primary antibodies in the experiments: Rat anti-HA monoclonal antibody (clone 3F10; Roche Diagnostics, Tokyo, Japan); mouse anti-SBP-tag monoclonal antibody (SB19-C4; Santa Cruz Biotechnology, Dallas, TX, USA); and rabbit anti-AIF polyclonal antibody (N1C1; GeneTex, Atlanta, GA, USA).

The following antibodies were used as secondary antibodies: goat anti-rabbit IgG-HRP antibody and goat anti-rat IgG-HRP antibodies (both from Santa Cruz Biotechnology); goat anti-mouse IgG-HRP antibody (GenScript); Alex-488 goat anti-rat IgG, Alex-488 donkey anti-mouse IgG antibody, and Alex-488 donkey anti-rabbit IgG antibody (from ThermoFisher Scientific). Streptavidin Mag Sepharose (GE Healthcare Life Sciences, Bjorkagatan, Uppsala, Sweden) was used for the SBP-pulldown experiment.

### Reagents

MG132, a proteasome inhibitor, was purchased from Cayman Chemical, Inc., (Ann Arbor, MI, USA); Staurosporine (STS), a non-selective protein kinase inhibitor widely used to induce both caspase-dependent and caspase-independent apoptosis^33^, was purchased from Focus Biomolecules, Inc. (Plymouth Meeting, PA,USA); MitoTracker Red CMXRos (ThermoFisher Scientific) was used to stain mitochondria. T lysis/wash buffer, which contained T buffer (20 mM HEPES–NaOH, pH 7.5, 150 mM NaCl, 2.5 mM MgCl_2_, 0.05% Tween-20, and 1 mM DTT) plus a protease inhibitor cocktail (Roche Diagnostics GmbH, Penzerg, Germany), was used for cell lysis and SBP pulldown. Biotin-elution buffer (2 mM Biotin in T buffer) was used to elute sepharose-binding proteins in the SBP-pulldown assay.

### Global analysis of E6-interacting proteins

293TT cells were cultured in a 150 mm dish one day before transfection. Then, 30 μg of plasmid (p16E6-SBP or pGFP-SBP) was suspended in 3 mL of phosphate-buffered saline (PBS), and then incubated with 120 μg (1 mg/mL) of PEI at room temperature for 20 min. The plasmid mixture was added to the 150 mm dish, and the medium was changed 4 h post transfection. Three dishes were used for each plasmid. The cells were washed twice with PBS at 24 h post-transfection and suspended in 2 mL of T lysis/wash buffer. The cell lysate was sonicated and centrifuged at 20,000×*g* at 4 °C for 30 min, and the supernatant was used for the SBP-pulldown assay. An appropriate amount of Streptavidin Mag Sepharose was washed three times with T lysis/wash buffer and blocked with 0.1% bovine serum albumin (BSA) in PBS at 37 °C for 1 h. After washing three times with T lysis/wash buffer, the Sepharose was incubated with the supernatant at 4 °C overnight with gentle agitation. The Sepharose was then washed with T lysis/wash buffer five times with gentle rotation (5 min per wash), and the Sepharose-binding proteins were eluted with biotin-elution buffer. The proteins were concentrated with a vapor centrifuge and stored at − 80 °C until LC–MS/MS analysis.

### Shotgun LC–MS/MS (liquid chromatograph mass spectrometer) analysis

LC–MS/MS analysis was performed using a TripleTOF MS (TripleTOF 5600 system; AB SCIEX, Foster City, CA, USA) and Analyst version 1.6 TF (AB SCIEX) coupled to a DiNa-AP (KYA Technologies, Tokyo, Japan) as previously described^61^. Prior to injection into the mass spectrometer, the tryptic digests were desalted using C18 membrane filters, and then loaded onto a reverse phase pre-column (HiQ sil C18W-3, 500 µm id × 1 mm; KYA Technologies) and resolved on a nanoscale HiQ sil C18W-3 (100 µm id × 10 cm; KYA Technologies) at a flow rate of 200 nL/min with a gradient of acetonitrile/0.1% (v/v) formic acid. Peptides were separated using a 65 min gradient from 5 to 45% solvent B (0.1% [v/v] formic acid/80% [v/v] acetonitrile). Solvent A was 0.1% (v/v) formic acid/2% (v/v) acetonitrile. The obtained MS and tandem-MS data were searched against the human protein sequences in the Swiss-Prot database (version Jan 2013, 20,233 sequences) using Protein Pilot software 4.0 (AB SCIEX, https://sciex.jp/support/knowledge-base-articles/can-protein-pilot-5-0-2-be-function-on-wwndows-7-64-bit-system).

### SBP-pulldown

293TT cells were transfected with an equal amount of each expression plasmid using PEI, and the cells were cultured for 24 h. Then, the cells were washed twice with PBS and suspended in T lysis/wash buffer. The cell lysate was sonicated and centrifuged at 20,000 × *g* for 30 min at 4 °C. The supernatant was used as the input for an SBP-pulldown assay. For the assay, an appropriate amount of Streptavidin Mag Sepharose was washed three times with T lysis/wash buffer and blocked with 0.1% BSA in PBS at 37 °C for 1 h. After the three washes, the Sepharose was incubated with the supernatant at 4 °C overnight with gentle agitation. Then, the Sepharose was washed with T lysis/wash buffer five times (5 min per wash), and the Sepharose-binding proteins were eluted with 1 × SDS loading buffer (50 mM Tris–Cl, pH 6.8, 2% sodium dodecyl sulfate [SDS], 0.1% bromophenol blue, 10% glycerol, and 100 mM dithiothreitol [DTT]) and incubated at 95 °C for 10 min. This sample was used for western blot analysis.

### Transfection and western blot analysis

For the transfection, 1 μg of plasmid DNA was suspended in 100 μL of PBS. Then, 4 μL of PEI (1 mg/mL) was added to the solution and gently mixed. This solution was incubated at room temperature for 20 min and then mixed with 293TT cells in a 12-well plate. Twenty-four hours after transfection, the cells were washed once with PBS and then mixed with 1 × SDS loading buffer and incubated at 95 °C for 10 min. In some experiments, 10 μM MG132 was added to the medium 12 h before cell harvest.

The cell lysate was loaded onto a SuperSep Ace 5–20% SDS PAGE (Wako, Tokyo, Japan) or a 10% SDS PAGE and electrophoresed in running buffer (25 mM Tris, 192 mM glycine, and 0.1% SDS) for 50 min at 180 V. The separated proteins were transferred to a nitrocellulose membrane using a Atto Western Blotting System (Tokyo, Japan) with EZ Fast Blot (Atto) for 15 min at 0.25 A per mini gel. The membrane was blocked with 5% skim milk at 37 °C for 20 min, and then washed five times with wash buffer (PBS with 0.5% Tween 20) at room temperature for 5 min with shaking. The membrane was incubated with primary antibody (1:3,000 dilution in PBS with 0.1% BSA) at 37 °C for 15 min. After washing with wash buffer at room temperature (five times for 5 min each), the membrane was incubated with a secondary antibody conjugated to horseradish peroxidase (HRP 1:3,000 dilution in 5% skim milk) at 37 °C for 15 min. After washing five times with the wash buffer at room temperature (5 min each), the protein was detected with SuperSignal West Femto Maximum Sensitivity Substrate (ThermoFisher Scientific), and analyzed by LAS-3000 with MultiGauge software Version 2.2 (Fujifilm, Tokyo, Japan, https://www.ualberta.ca/biological-sciences/media-library/mbsu/fla-5000/mulitgauge20.pdf).

### Immunostaining

U2OS cells were transfected with the expression plasmids using PEI for 20 h. Then, the cells were washed twice with PBS and fixed/permeabilized with cold methanol/acetone (1:1) at 4 °C for 5 min. The cells were washed three times with PBS, blocked with 3% BSA in PBS for 30 min at room temperature, and then incubated with rabbit anti-human AIF antibody, rat anti-HA-tag antibody, and/or mouse anti-SBP antibody (1:500 dilution in PBS containing 1% BSA) for 1 h at room temperature. Next, the cells were washed with PBS containing 0.1% BSA six times (5 min each). Then, the cells were incubated with a fluorescence-labeled secondary antibody (1:500 dilution in PBS containing 1% BSA). In some experiments, the cells were stained with 250 μM MitoTracker Red CMXRos (ThermoFisher Scientific) in culture medium at room temperature for 5 min before fixation and permeabilization. The plasmid transfection efficacy was approximately 30%, and more than 90% of co-transfected cells were double positive, with two co-transfected plasmids. For each immunostaining, 50 exogenous protein-positive cells were evaluated, and most showed similar staining results. Figure 4 represents the typical staining that was observed for each transfected cell. Protein localization was determined by confocal microscopy (FV1000-D IX81; Olympus, Tokyo, Japan).

### Nuclei isolation

Nuclei were isolated from HeLa cells using a nucleus isolation kit (NUC-1KT; Sigma) according to the manufacturer’s instructions. Briefly, confluent HeLa cells cultured in a 100 mm dish were washed twice with PBS, and then 4 mL of cold Nuclei EZ lysis buffer was added to the plate. Cells were harvested with a cell scraper. After brief vortexing, the cells were incubated on ice for 5 min, and the nuclei were collected by centrifugation at 500 × *g* for 5 min. The pelleted nuclei were resuspended in 4 mL of cold Nuclei EZ lysis buffer, incubated on ice for 5 min, and then centrifuged at 500×*g* for 5 min. The pellet was resuspended in 200 μL of cold Nuclei EZ storage buffer and stored at − 80 °C until use.

### Preparation of cytosolic extracts

Cytosolic extracts were prepared as previously described^56^. Briefly, 293TT cells were transfected with the recombinant expression plasmids pΔ102AIF, p16E6, and p6E6. Two days after transfection, the cells were washed twice with PBS and harvested with a cell scraper. The cells were suspended in fractionation buffer (250 mM sucrose, 20 mM HEPES, pH 7.3, 10 mM KCl, 1.5 mM MgCl_2_, 1 mM EDTA, 1 mM EGTA, 1 mM DTT, and proteinase inhibitor cocktail) and sonicated (Bioruptor UCD-250; Cosmo Bio, Tokyo, Japan) for 15 min with intervals of 30 s on and 30 s off at 4 °C. The cell lysates were centrifuged at 20,000×*g* for 30 min at 4 °C, and the supernatant, as a cytosolic extract, was stored at –80 °C until use. The cytosolic extracts from normal cells were used as a control.

### Chromatin degradation assay

The chromatin degradation assay was performed as previously described^56^. Briefly, 100 μL of cytosolic extract (100 μg of total protein) was incubated with 100,000 nuclei for 90 min at 37 °C. After incubation, 400 μL of fractionation buffer containing 4 μg/mL propidium iodide was added to each sample, and then the samples were analyzed by flow cytometry (BD FACSCanto II; BD Bioscience, East Rutherford, NJ, USA) using BD FACSDiva software (version 6.1.3, https://www.bdbiosciences.com/en-us/instruments/research-instruments/research-software/flow-cytometry-acquisition/facsdiva-software). Thirty thousand of nuclei were counted for each sample and nuclei containing degraded chromatin were quantified as sub-G1 PI positive.

### Cell apoptosis induction

MEF, MEF-16E6, and MEF-6E6 cells were transfected with siControl or siAIF twice at a 12 h interval. Forty-eight hours after transfection, the cells were stimulated with STS for 4 h, and then harvested and washed with PBS. Apoptosis was detected by the FITC-Annexin V apoptosis detection kit with propidium iodide (PI; Biolegend, San Diego, CA, USA) according to the manufacturer’s instructions. Cell apoptosis was recorded with a BD FACSCanto II (BD Bioscience).

### Data analysis

All data were expressed as means and standard deviation (SD). Statistical analyses were performed by the Mann–Whitney *U* test for two group comparison and by Krushal-Wallis test with Steel–Dwass test for multi-group comparison using Microsoft Excel software (Bell Curve). A *p* value less than 0.05 was considered statistically significant.

## Supplementary information

Supplementary Table 1.

Supplementary Figures.

## Data Availability

The materials and information are available from the corresponding author on reasonable request.
