# Supplementary material for: The human papillomavirus E6 protein targets apoptosis-inducing factor (AIF) for degradation
Source: Sci Rep. 2020 Aug 26;10:14195. doi: 10.1038/s41598-020-71134-3 (PMC7450093; doi:10.1038/s41598-020-71134-3)
Supplement: Supplementary file 1 — Supplementary Table 1. [file 41598_2020_71134_MOESM1_ESM.pdf]

The human papillomavirus E6 protein targets apoptosis-inducing factor (AIF) for degradation

Masaru Shimada[1]*, Akio Yamashita[2], Manami Saito[1], Motohide Ichino[3], Takao Kinjo[4], Nobuhisa Mizuki[5], Dennis M. Klinman[6] & Kenji Okuda[1]

[1]Department of Molecular Biodefense Research, Yokohama City University, Yokohama 236-0004, Japan
[2]Department of Molecular Biology, Yokohama City University, Yokohama 236-0004, Japan
[3]Department of Immunology, Yokohama City University, Yokohama 236-0004, Japan
[4]Division of Morphological Pathology, Department of Basic Laboratory Sciences, School of Health Sciences, University of the Ryukyus, Okinawa 903-0215, Japan
[5]Department of Ophthalmology and Visual Science, Yokohama City University, Yokohama 236-0004, Japan
[6]Frederick National Laboratory for Cancer Research, National Cancer Institute, NIH, Frederick, MD 21702, USA

Supplementary Table S1

SYNTHESIZED GENE	SEQUENCE
Synthetic, codon-optimized HPV16 E6	ATGCATCAGAAACGGACCGCAATGTTTCAGGATCCTCAGGAGCGGCCCCGCAAGCTCCCC<br>CAGCTGTGTACCGAGCTGCAGACAACCATCCACGACATCATACTGGAATGTGTTTACTGCA<br>AACAACAGCTCCTCAGAAGAGAAGTTTATGATTTTGCCTTCAGAGACCTGTGCATTGTTTA<br>CAGGGATGGTAATCCGTATGCCGTCTGCGATAAGTGTCTGAAATTCTACTCCAAGATCAGC<br>GAGTACCGGCACTACTGTTACAGCCTGTACGGGACCACACTCGAACAACAGTACAACAAG<br>CCACTCTGTGACCTGCTTATCAGGTGTATTAACTGTCAGAAACCCCTGTGCCCAGAAGAGA<br>AGCAGCGGCATCTGGACAAGAAGCAGCGCTTCCACAATATTAGAGGCCGCTGGACTGGCA<br>GGTGTATGTCATGCTGTAGATCCAGTCGCACGAGACGGGAAACTCAATTG
Synthetic, codon-optimized HPV6 E6	ATGGAATCTGCCAATGCCTCAACGAGTGCTACCACCATAGACCAACTGTGCAAGACCTTCA<br>ATCTGTCCATGCACACCCTTCAGATTAACTGCGTCTTTTGCAAGAATGCACTCACAACTGC<br>CGAGATCTACAGCTACGCCTACAAACAGCTGAAAGTGCTGTTTAGGGGTGGCTATCCTTAT<br>GCCGCTTGTGCATGCTGTCTGGAGTTCCATGGCAAGATCAACCAGTACCGACATTTCGAC<br>TATGCGGGATATGCAACAACCGTAGAGGAAGAGACAAAGCAGGACATTCTGGATGTTCTC<br>ATTCGGTGCTACTTGTGTCACAAGCCATTGTGCGAAGTGGAAAAGGTGAAACACATCCTTA<br>CTAAAGCTCGCTTTATCAAGCTGAACTGTACTTGGAAAGGGAGATGTCTCCATTGCTGGAC<br>AACGTGTATGGAGGATATGCTGCCCtga

OLIGONUCLEOTIDES	SEQUENCE
pCASSG (EcoR I) Forward	ACTCCTCAGGTGCAGGCTGCCTATCAGAA
pCAGGS (EcoR I) Revise	TTTGCCAAAATGATGAGACAG

RECOMBINANT DNA	TRANSGENE(S)	SOURCE	IDENTIFIER
Plasmid: pCAGGS-AIF-HA	AIF-HA	This paper	N/A
Plasmid: pCAGGS-GFP-HA	GFP-HA	This paper	N/A
Plasmid: pCAGGS-HPV16E6-SBP	HPV16E6-SBP	This paper	N/A
Plasmid: pCAGGS-GFP-SBP	GFP-SBP	This paper	N/A
Plasmid: pCAGGS-△54AIF-HA	Ubi-54AIF-HA	This paper	N/A
Plasmid: pCAGGS-△102AIF-HA	Ubi-102AIF-HA	This paper	N/A
Plasmid: pCAGGS-HPV6E6-SBP	HPV6E6-SBP	This paper	N/A
Plasmid: pCAGGS-△1	Ubi-AIF(FAD1)-GFP-HA	This paper	N/A
Plasmid: pCAGGS-△2	Ubi-AIF(NADH)-GFP-HA	This paper	N/A
Plasmid: pCAGGS-△3	Ubi-AIF(FAD2)-GFP-HA	This paper	N/A
Plasmid: pCAGGS-△4	Ubi-AIF(C terminal)-GFP-HA	This paper	N/A
Plasmid: pCAGGS-△5	Ubi-AIF(NADH-FAD2-C terminal)-HA	This paper	N/A
Plasmid: pCAGGS-△6	Ubi-AIF (FAD1-FAD2-C terminal)-HA	This paper	N/A
Plasmid: pCAGGS-△7	Ubi-AIF (FAD1-NADH-C terminal)-HA	This paper	N/A
Plasmid: pCAGGS-△8	Ubi-AIF (FAD1-C terminal)-HA	This paper	N/A
Plasmid: pCAGGS-△9	Ubi-AIF (NADH-C terminal)-HA	This paper	N/A
Plasmid: pCAGGS-△10	Ubi-AIF (NADH-FAD2-C terminal)-HA	This paper	N/A
Plasmid: pCAGGS-△11	Ubi-AIF (FAD1-NADH)-HA	This paper	N/A
Plasmid: pCAGGS-△12	Ubi-AIF (FAD1-NADH-FAD2)-HA	This paper	N/A
Plasmid: pCAGGS-AIF	AIF	This paper	N/A
Plasmid: pCAGGS-GFP	GFP	This paper	N/A
Plasmid: pCAGGS	Non	This paper	N/A
Plasmid: HPV16E6	HPV16E6	This paper	N/A
Plasmid: HPV6E6	HPV6E6	This paper	N/A
