# Supplementary material for: The human papillomavirus E6 protein targets apoptosis-inducing factor (AIF) for degradation
Source: Sci Rep. 2020 Aug 26;10:14195. doi: 10.1038/s41598-020-71134-3 (PMC7450093; doi:10.1038/s41598-020-71134-3)

The human papillomavirus E6 protein targets apoptosis-inducing factor (AIF) for degradation

Masaru Shimada[1]*, Akio Yamashita[2], Manami Saito[1], Motohide Ichino[3], Takao Kinjo[4], Nobuhisa Mizuki[5], Dennis M. Klinman[6] & Kenji Okuda[1]

[1]Department of Molecular Biodefense Research, Yokohama City University, Yokohama 236-0004, Japan
[2]Department of Molecular Biology, Yokohama City University, Yokohama 236-0004, Japan
[3]Department of Immunology, Yokohama City University, Yokohama 236-0004, Japan
[4]Division of Morphological Pathology, Department of Basic Laboratory Sciences, School of Health Sciences, University of the Ryukyus, Okinawa 903-0215, Japan
[5]Department of Ophthalmology and Visual Science, Yokohama City University, Yokohama 236-0004, Japan
[6]Frederick National Laboratory for Cancer Research, National Cancer Institute, NIH, Frederick, MD 21702, USA

Figure S1. Binding of E6 to AIF, Related to Figure 2

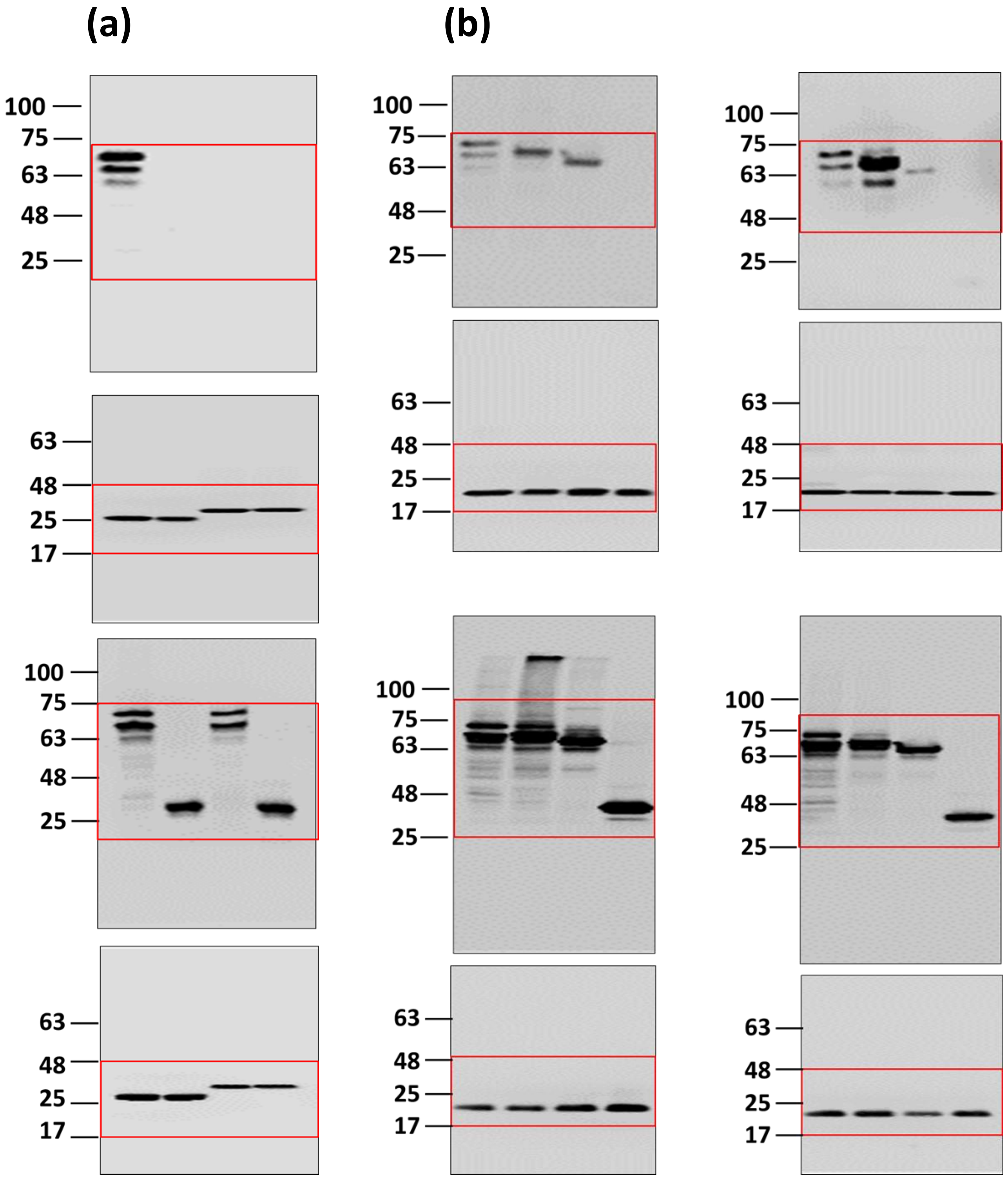

Figure S2. E6 binds to the FAD domain of AIF. Related to Figure 3b

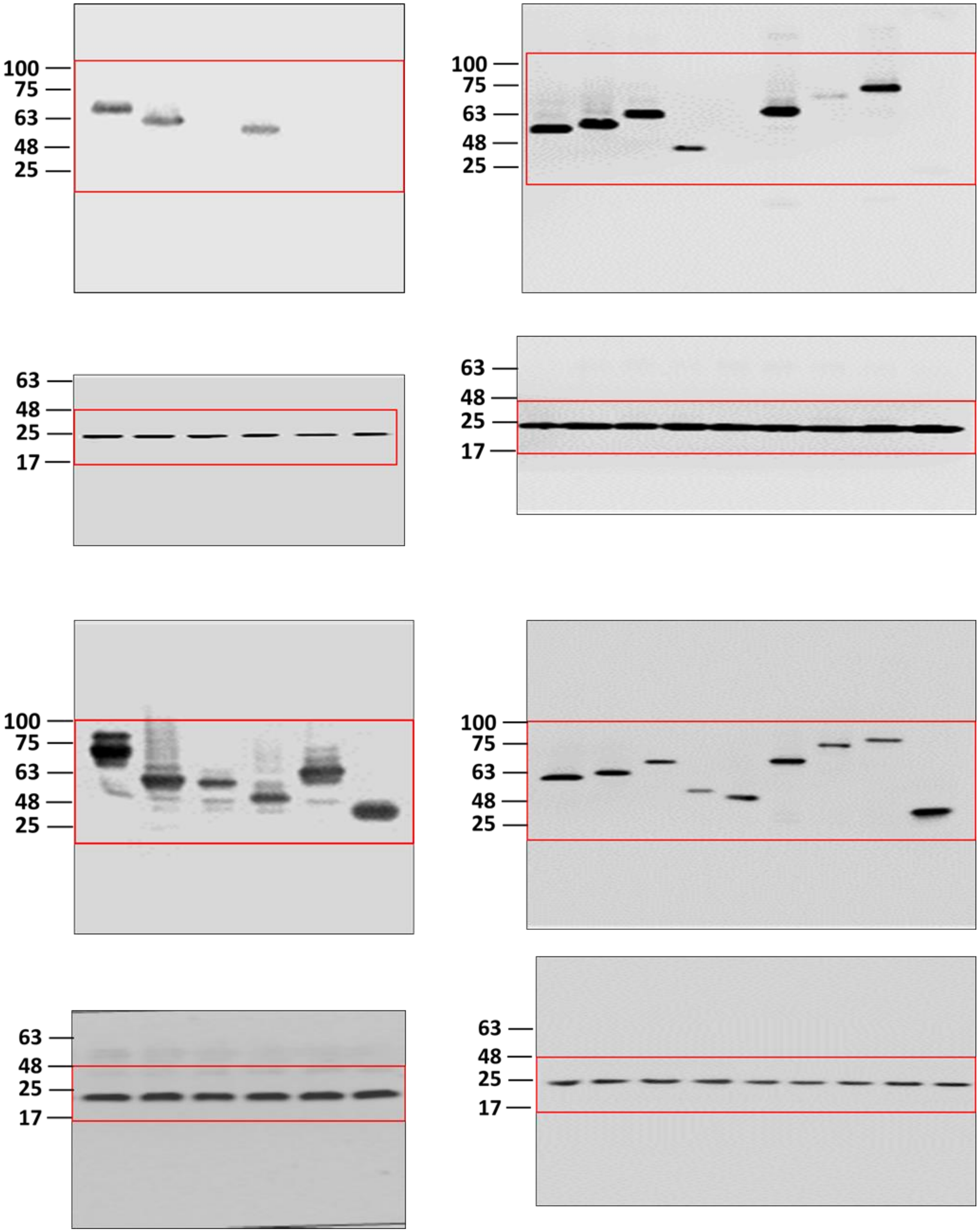

Figure S3. Proteasomal degradation of AIF induced by E6. Related to Figure 5

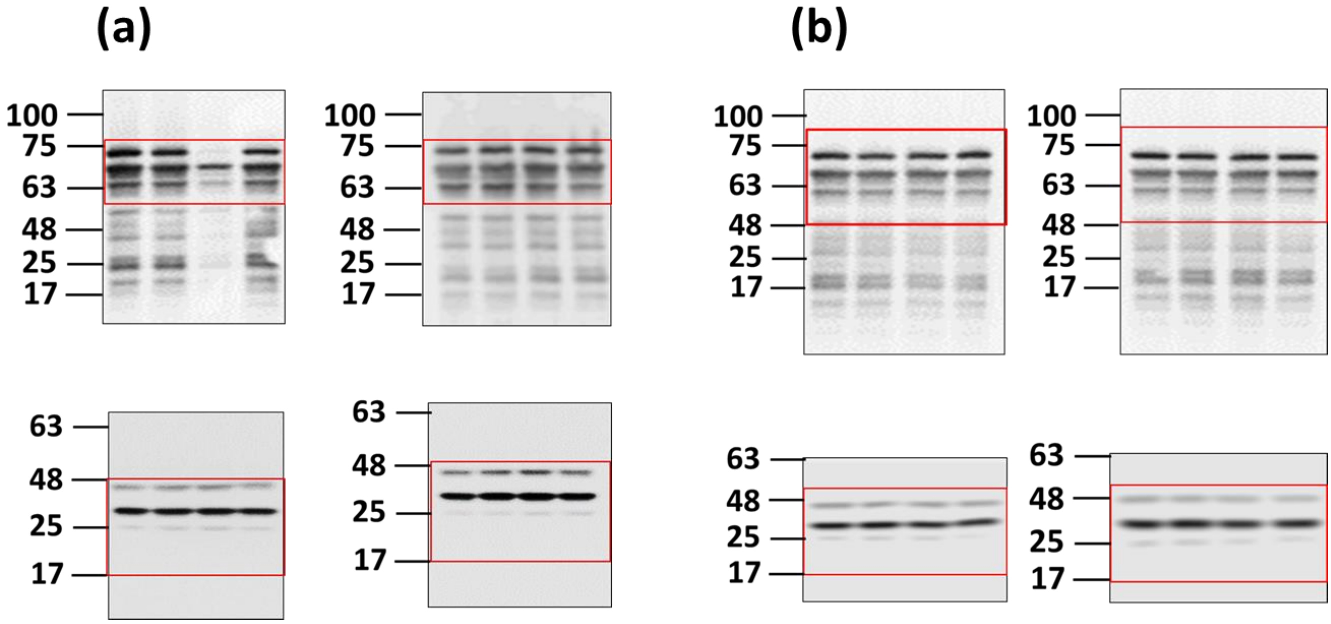

Figure S4. Alignment of HPV E6 proteins among genotype. Alignment was performed using the ClustalW software.

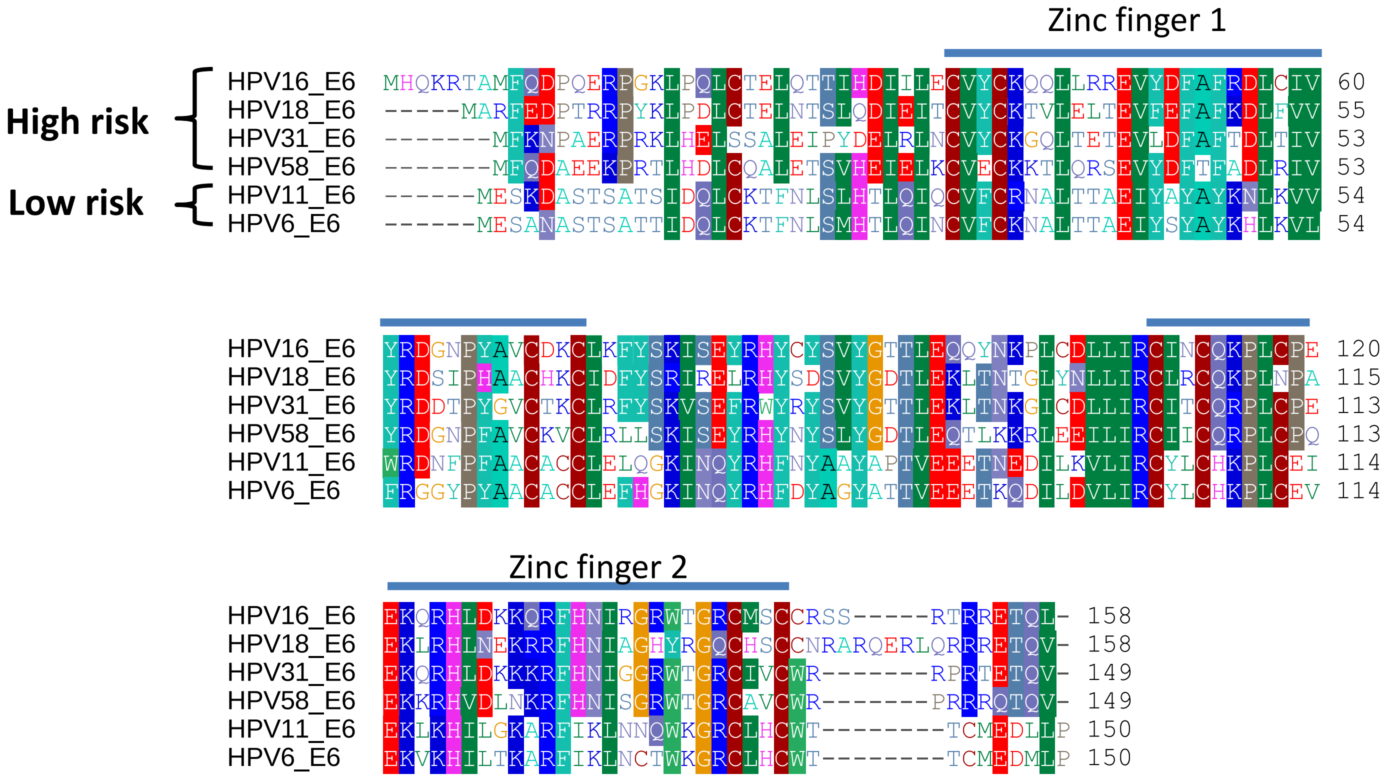

Supplement: Supplementary file 2 — Supplementary Figures. [file 41598_2020_71134_MOESM2_ESM.pdf]
